# Impact of Skeletal Muscle-related Parameters on Survival in Patients with Advanced Pancreatic Cancer Treated with Gemcitabine plus Nab-paclitaxel as First-line Chemotherapy

**DOI:** 10.7150/jca.126673

**Published:** 2026-02-26

**Authors:** Nanako Matsuo, Toshifumi Yamaguchi, Hiroki Yukami, Hiroyuki Kodama, Takako Ikegami, Toru Kadono, Shin Kameisihi, Dai Okemoto, Elham Fakhrejahani, Hiroki Nishikawa

**Affiliations:** 1 Cancer Chemotherapy Center, Osaka Medical and Pharmaceutical University Hospital, Osaka, Japan; 2 Second Department of Internal Medicine, Osaka Medical and Pharmaceutical University, Osaka, Japan; 3 Department of Gastrointestinal Oncology, National Cancer Center Hospital, Tokyo, Japan; 4 Department of Gastrointestinal Oncology, National Cancer Center Hospital East, Kashiwa, Japan; 5 Kyoto Breast Cancer Research Network

**Keywords:** Advanced pancreatic cancer, Gemcitabine plus nab-paclitaxel, Psoas muscle, Fat deposition, Prognosis

## Abstract

**Background:**

Sarcopenia, defined as a reduction in muscle mass assessed using scales such as the psoas muscle mass index (PMI), is accompanied by decreased muscle strength or physical function. However, sarcopenia's effect in patients with pancreatic cancer (PC) receiving chemotherapy remains unclear. In addition, recent international studies have demonstrated that intramuscular fat infiltration, assessed using parameters such as FRPM, is associated with poor prognosis across various malignancies. However, evidence regarding its prognostic significance in pancreatic cancer remains limited. We aimed to evaluate the relationship between sarcopenia and the prognosis of patients with PC receiving palliative chemotherapy.

**Methods:**

We retrospectively reviewed patients diagnosed with unresectable PC who received gemcitabine plus nab-paclitaxel (GnP) as the first-line therapy at our hospital between 2018 and 2021. We calculated PMI, defined as the sum of the bilateral psoas muscle mass at the lumbar three (L3) level and FRPM, defined as the sum of areas within the psoas muscles corresponding to fat at the L3 level from Vincent® on the CT images. We compared the overall survival (OS) between the PMI-high and PMI-low groups and the FRPM-high and FRPM-low groups.

**Results:**

Of 46 patients, 37 were eligible. Eighteen (49%) and 19 (51 %) patients were classified into PMI-high and PMI-low groups, respectively. Twenty (54%) and 17 patients (46%) were classified into FRPM-high and FRPM-low groups, respectively. The median OS was 16.4 months in PMI-high and 8.7 months in PMI-low groups (hazard ratio [HR]: 0.45, 95% confidence interval [CI]: 0.23-0.90, P < 0.01). The median OS was 15.6 months in FRPM-low and 8.5 months in FRPM-high groups (HR: 0.36, 95% CI: 0.18-0.76, P < 0.01). In multivariate analysis, the presence of ascites (P < 0.01), PMI-low (P = 0.02), and FRPM-high (P = 0.03) were independent adverse prognostic factors for OS.

**Conclusion:**

Muscle-related parameters may be independent indicators of poor prognosis in patients with PC treated with first-line GnPs.

## Introduction

Pancreatic cancer (PC) is the fourth leading cause of cancer-related death in Japan, and its incidence and mortality rates are increasing steadily 1. PC is a lethal condition of the digestive system, and patients have a poor prognosis. Incidence and mortality rates of PC have increased over the past decades 2-4. Despite advances in systemic chemotherapy and supportive care, the prognosis of patients with PC remains extremely poor, with a 5-year overall survival (OS) rate of approximately 10% 5. In patients with stage IV PC, the 5-year survival rate drops to < 2%, underscoring the disease's aggressive nature and the urgent need to identify clinical and biological factors that can guide treatment decisions and predict prognosis 6. Among the standard first-line chemotherapy regimens for unresectable PC, gemcitabine combined with nanoparticle albumin-bound paclitaxel (nab-paclitaxel) has shown a survival benefit over gemcitabine monotherapy and is now widely used in clinical practice 7. However, the therapeutic response to this regimen substantially varies among patients, suggesting that the tumor burden alone is insufficient to predict treatment outcomes. In recent years, host-related factors, including nutritional status, body composition, and systemic inflammation, have gained research traction. These have been shown to influence treatment tolerance and survival outcomes. Sarcopenia, defined as the progressive loss of skeletal muscle mass, strength, or physical performance, is highly prevalent in patients with advanced cancer, particularly in those with PC 8-12. These patients are often older and are commonly affected by cancer cachexia, reduced physical activity, and insufficient oral intake. Sarcopenia is associated with increased chemotherapy-related toxicity, early treatment discontinuation, impaired antitumor immunity, and poorer OS 13-16. However, its prognostic value in patients with PC undergoing palliative chemotherapy, including those treated with gemcitabine and nab-paclitaxel (GnP), remains unclear. In addition to skeletal muscle quantity, muscle quality, particularly the degree of intramuscular fat infiltration, or myosteatosis, has been recently acknowledged as a clinically relevant indicator of muscle function and metabolic health 17. Myosteatosis is associated with insulin resistance, inflammation, and frailty and may contribute to poor treatment outcomes in patients with cancer. Specifically, the psoas muscle mass index (PMI), which reflects muscle quantity, and the fat ratio within the psoas muscle (FRPM), which reflects muscle quality, have emerged as objective and quantifiable markers of sarcopenia in clinical settings 18-21. We previously reported the prognostic impact of FRPM in patients with stage IV gastric cancer who were undergoing systemic chemotherapy 22.

Systemic inflammation is increasingly being recognized as a hallmark of cancer progression and is associated with malnutrition and muscle wasting. In this context, inflammation-based biomarkers, such as the Glasgow Prognostic Score (GPS) and neutrophil-to-lymphocyte ratio (NLR), have emerged as simple and cost-effective prognostic tools. GPS, which integrates serum C-reactive protein (CRP) and serum albumin levels, reflects both systemic inflammation and nutritional status, while NLR provides insight into the balance between protumor neutrophilic activity and antitumor lymphocyte-mediated immune surveillance. Both indices are correlated with poor survival in various malignancies, including PC 22-2.

We investigated the prognostic significance of sarcopenia and intramuscular fat infiltration in patients with unresectable PC who underwent GnP therapy. We focused on objective indices, such as PMI and FRPM, and examined their association with treatment efficacy and survival outcomes. We explored their relationship with systemic inflammatory markers, such as GPS and NLR.

## Materials and Methods

### Patients and methods

We retrospectively reviewed the medical records of patients with histologically confirmed advanced PC at the Osaka Medical and Pharmaceutical University, Osaka, Japan. Inclusion criteria were as follows: patients who received GnP, no systemic chemotherapy before receiving GnP after the diagnosis of inoperable PC, Eastern Cooperative Oncology Group performance status (ECOG-PS) of 0-2, and histologically confirmed PC.

Between April 2018 and March 2021, 46 patients diagnosed with unresectable PC were identified in our medical records, of whom 37 underwent first-line chemotherapy with GnP. They were eligible for our analysis. Five patients who received other treatments and four with immature dates were excluded from the analysis. The primary endpoint was OS. Factors relevant to OS were retrospectively analyzed by univariate and multivariate analyses. The Ethics Committee of Osaka Medical and Pharmaceutical University Hospital (approval number, 2024-057) approved the study. The requirement for informed consent from each patient was waived because of the study's retrospective nature.

Age, sex, ECOG-PS, body mass index (BMI), and laboratory data were extracted from medical records, and prognostic indicators reflecting inflammation and cachexia, GPS (GPS = 0 was assigned when CRP levels were < 1.0 mg/dL and serum albumin levels were > 3.5 g/dL; GPS = 1 was assigned when CRP was > 1.0 mg/dL or serum albumin levels were < 3.5 g/dL, and GPS = 2 was assigned when CRP levels were > 1.0 mg/dL and serum albumin levels were < 3.5 g/dL). NLR was calculated from laboratory data. The presence or absence of ascites was determined from computed tomography (CT) images.

### Treatment

Almost all patients were treated with nab-paclitaxel (125 mg/m^2^) and gemcitabine (1,000 mg/m^2^) as the first-line chemotherapy on days 1, 8, and 15 every 4 weeks, constituting one treatment cycle. In some patients, doses were reduced based on certain factors, such as age and ECOG-PS. First-line chemotherapy was discontinued in patients who developed progressive disease (PD). After initiating treatment, patients underwent careful follow-up and imaging, and tumor marker measurements. Patients with PD received a second-line chemotherapy regimen or adequate supportive care. The start date of the follow-up was set as the date of initiation of first-line chemotherapy. The end date of the follow-up was set as the final follow-up date in June 2021 or the date of death.

### Measurement of skeletal muscle mass

PMI was defined as the sum of bilateral psoas muscle mass calculated by Vincent® (SYNAPSEVINCENT, Fuji Film Medical Corporation, Tokyo, Japan) at the lumbar three (L3) level on the CT images (at the time of initial chemotherapy), divided by height squared (cm^2^/m^2^). We proposed FRPM at the L3 level (at the time of initial chemotherapy) based on our previous findings and calculated it as follows: fat mass within the bilateral psoas muscle at the L3 level was calculated using Vincent's formula (the sum of the areas within the left and right psoas muscles corresponding to fat (cm^2^)) divided by bilateral psoas muscle mass calculated using Vincent's formula (cm^2^) × 100 (%) 20. The CT density for fat was defined between -200 Hounsfield Units (HU) and -50 HU. To minimize measurement bias, a single well-trained researcher (T. I.) identified and measured the psoas muscle mass and fat within the psoas muscle.

### Statistical analysis

Continuous variables between two groups were compared by Student's *t*-test or Mann-Whitney U test, as appropriate. Categorical variables between groups were compared by the Pearson χ^2^ test. Continuous parameters were divided into two groups at the median value and transformed into nominal variables to analyze the significance of the prognostic parameters. Continuous analyses of PMI and FRPM were also considered; however, because of potential overfitting and instability of estimates, the dichotomized approach was prioritized. The cutoff value for NLR (3.44) was determined based on the median value within our cohort. Currently, there is no standardized cutoff value for NLR, and previous studies have reported substantial heterogeneity in cutoff definitions, with some studies adopting the median value as the cutoff. Therefore, we adopted this approach in the present study 25. The Kaplan-Meier method and log-rank test were used to estimate the cumulative OS rate. A Cox proportional hazard model was used for multivariate analysis of parameters with a P-value < 0.05 in the univariate analysis. The observation period was defined as the time interval between the date of initial chemotherapy and the date of death or the last date of confirmed survival. For data presentation, n (%) or median (range) was used unless stated otherwise. JMP ver. 15 (SAS Institute Inc., Cary, NC, USA) was used for statistical analysis, and a P value < 0.05 was considered statistically significant.

## Results

### Baseline patient characteristics

The baseline characteristics of all study participants (n=37, 20 men and 17 women, median (range) age =70 (49-86) years) are summarized in Table 1. The median (range) BMI was 20.0 (14.2-32.9) kg/m^2^. An ECOG PS of 0 was found in 11 patients (29.7%), 1 in 25 (67.6%), and 2 in 1 (2.7%). Twenty-six patients (70.3%) had no ascites, 8 (21.6%) had mild ascites, 2 (5.4%) had moderate ascites, and 1 (2.7%) had severe ascites. The median (range) FRPM was 2.55 (0.05-9.01) %. Twenty patients with FRPM ≥ 2.55% were categorized into the FRPM-high group, and the remaining 17 were in the FRPM-low group. The median (range) PMI in male and female patients was 3.92 (2.11-6.73) cm^2^/m^2^ and 3.57 (2.17-4.49) cm^2^/m^2^. Ten male patients with PMI ≥ 3.92 cm^2^/m^2^ and 8 female patients with PMI ≥ 3.57 cm^2^/m^2^ constituted the PMI-high group, and the remaining 19 patients were in the PMI-low group.

### Initial systemic chemotherapy

In terms of initial chemotherapy, GnP was administered in 37 patients, chemoradiotherapy in 3, oxaliplatin and irinotecan plus fluorouracil (FOLFIRINOX) in 2, and data were insufficient in 4 patients. The median duration of GnP therapy was 118 days, and the median initial treatment doses were 123 mg/m²/day for nab-paclitaxel and 996 mg/m²/day for gemcitabine.

### Cumulative OS rate according to the PMI and FRPM

**Figure 1:**
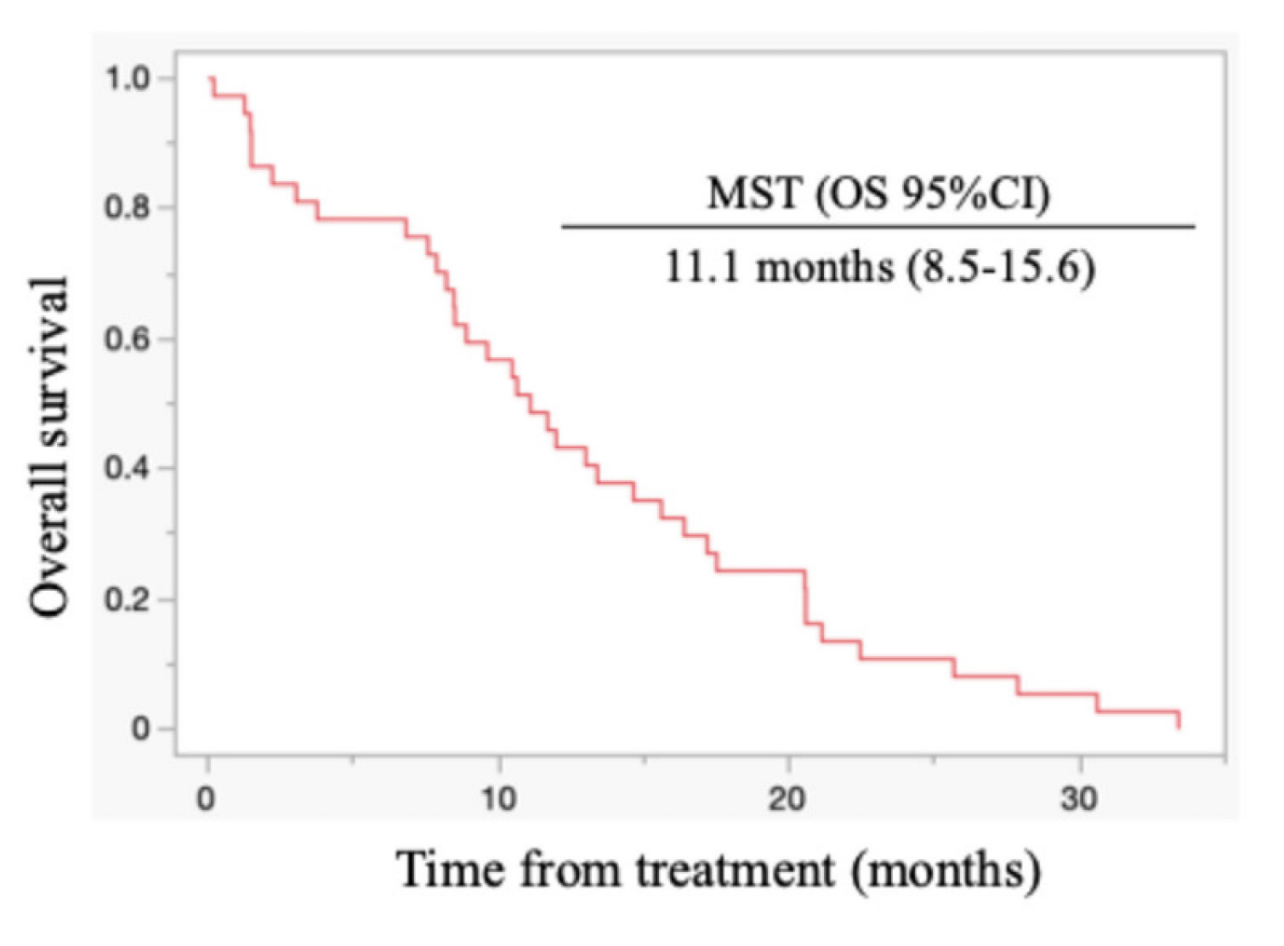
Kaplan-Meier curves for all cases. MST: median survival time; OS: overall survival; CI: confidence interval.

**Figure 2:**
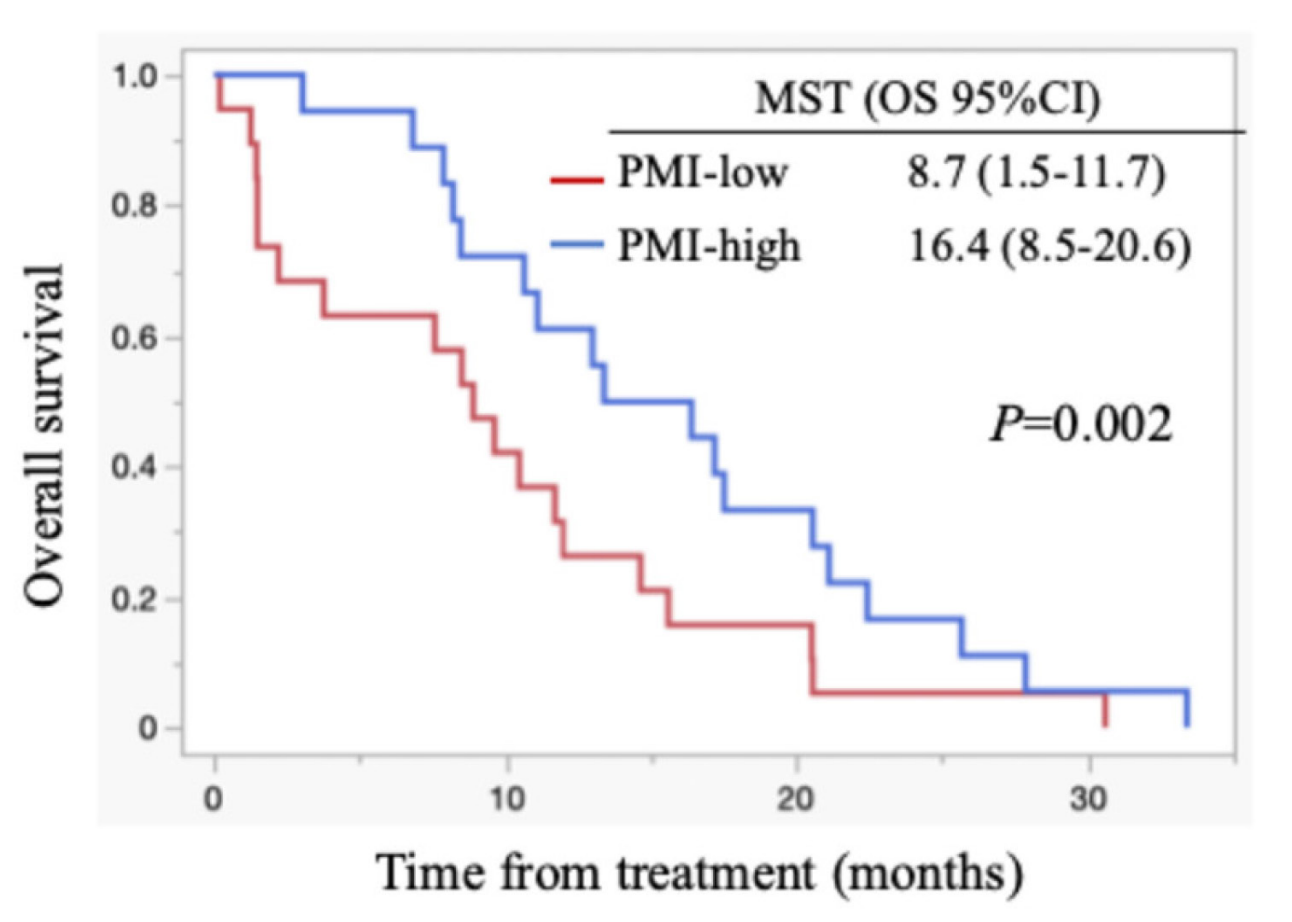
Kaplan-Meier curves stratified by PMI for all cases. MST: median survival time; OS: overall survival; CI: confidence interval.

**Figure 3:**
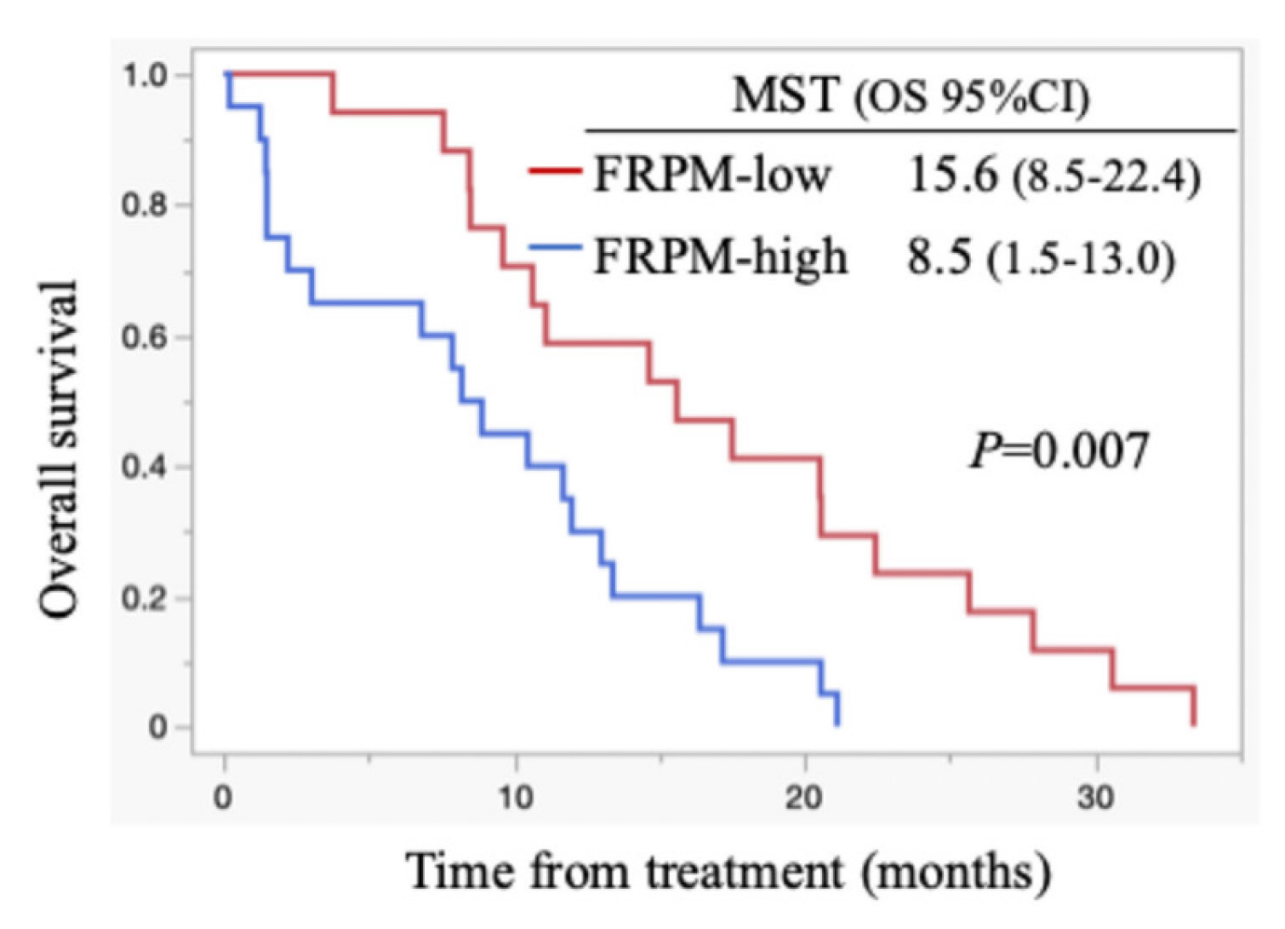
Kaplan-Meier curves stratified by FRPM for all cases. MST: median survival time; OS: overall survival; CI: confidence interval.

All patients died during the follow-up period, and these were PC-related deaths. The median OS for all cases was 11.1 months (95% confidence interval (CI): 8.5-15.6 months) (Figure 1). Regarding post-progression management, 24 patients (64.9%) received second-line chemotherapy after disease progression: 13 patients were treated with nanoliposomal irinotecan plus 5-fluorouracil, 2 with FOLFOX, 6 with S-1, and 3 with irinotecan monotherapy. The remaining 13 patients did not receive second-line therapy. Information on whether patients underwent nutritional counselling or rehabilitation during treatment was unavailable. The median progression-free survival (PFS) was 5.5 months (95% CI: 4.4-6.9 months), and the 6-month PFS rate was 45.9 %. The objective response rate (ORR) was 21.6 % (8/37). FRPM-high was significantly associated with shorter PFS (P = 0.0023), whereas no significant correlations were found between PMI and PFS (P = 0.2927), or between PMI/FRPM and ORR (P = 0.3743 and P = 0.5862, respectively). The median OS was 16.4 months in PMI-high and 8.7 months in PMI-low (HR: 0.45, 95% CI: 0.23-0.90, P = 0.002) (Figure 2). The median OS was 15.6 months in FRPM-low and 8.5 months in FRPM-high (HR: 0.36, 95% CI: 0.18-0.76, P = 0.007) groups (Figure 3). The 1-year cumulative OS rates of patients in PMI-high and PMI-low groups were 58% and 26%, respectively. The 1-year cumulative OS rates in the FRPM-high and FRPM-low groups were 43% and 55%, respectively.

### Survival according to FRPM and PMI in males and females

**Figure 4:**
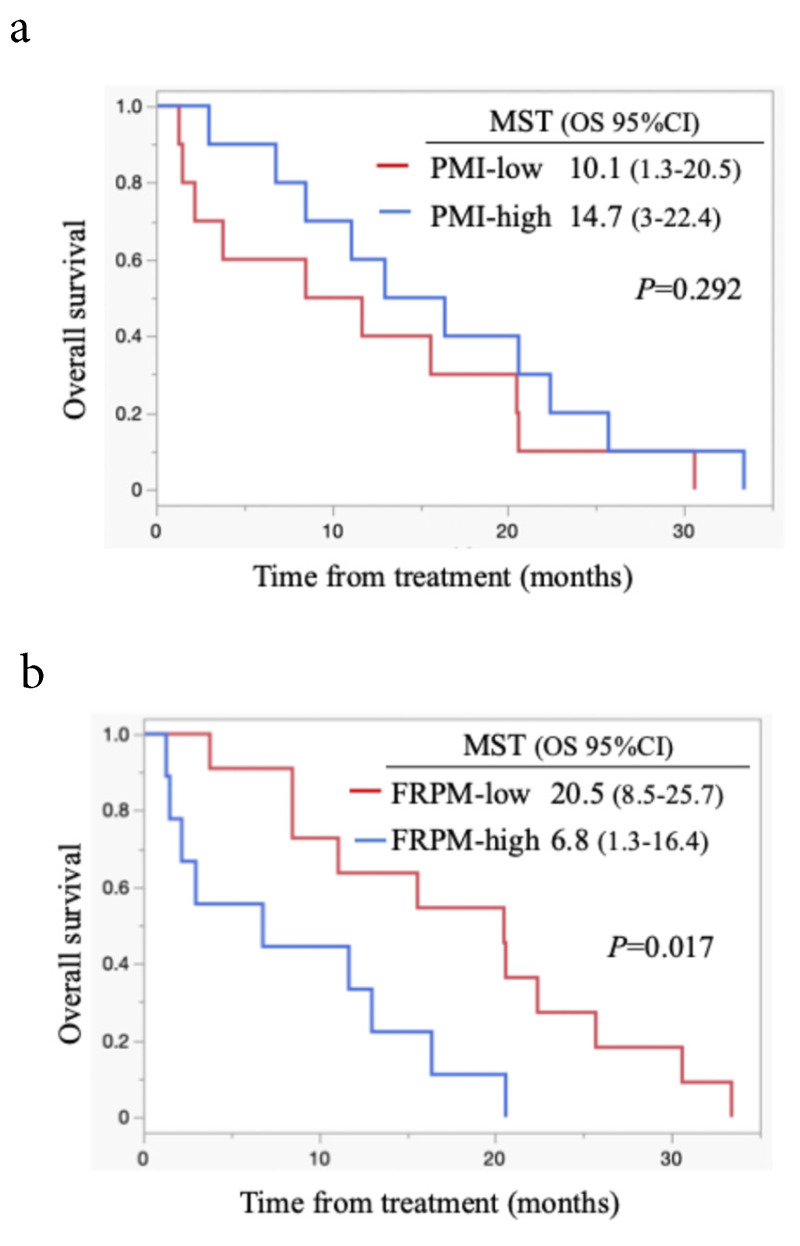
Kaplan-Meier curves stratified by PMI (4a) and FRPM (4b) in males. MST: median survival time; OS: overall survival; CI: confidence interval.

**Figure 5:**
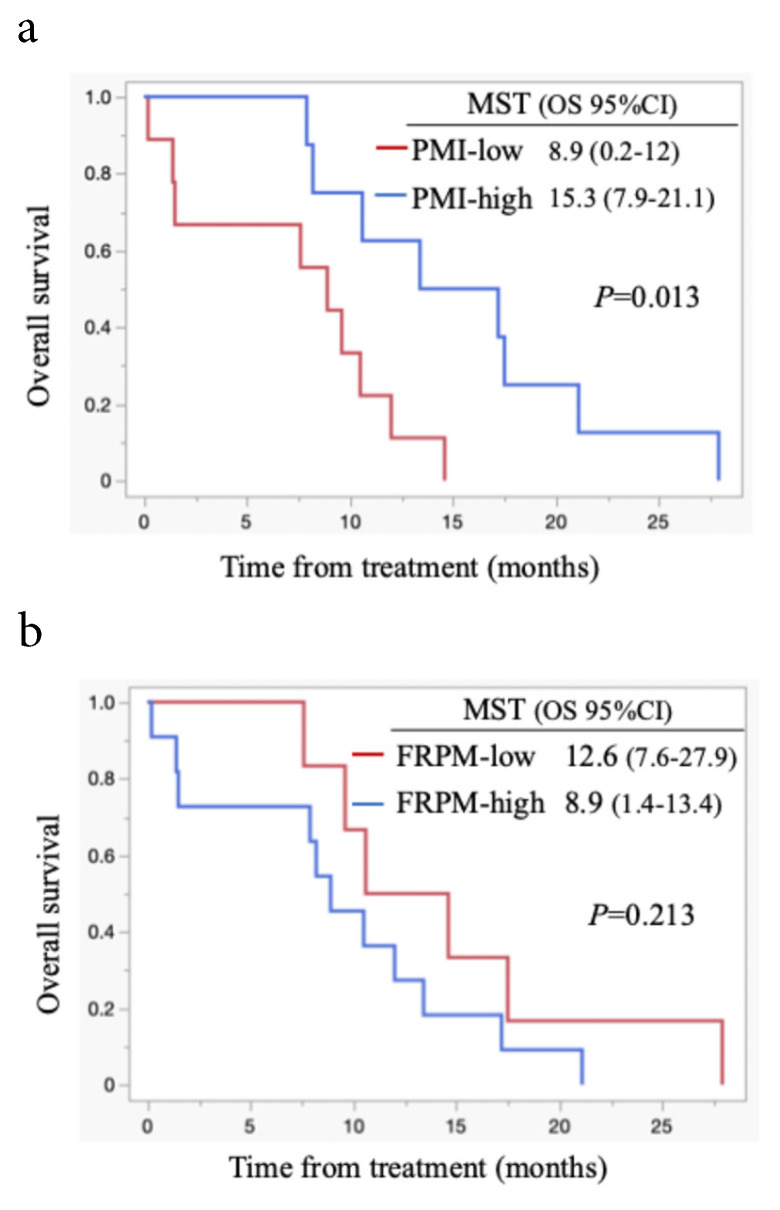
Kaplan-Meier curves stratified by PMI (5a) and FRPM (5b) in females. MST: median survival time; OS: overall survival; CI: confidence interval.

Survival analysis according to PMI and FRPM in males is shown in Figures 4a and 4b. In males, survival between the PMI-high and PMI-low groups did not differ significantly (P = 0.292, Figure 4a). Patients with FRPM-low showed better survival than those with FRPM-high (P = 0.017; Figure 4b). In females, although patients in the PMI-high group showed significantly better survival than those in the PMI-low group (P = 0.013, Figure 5a), the difference in survival did not reach statistical significance (P = 0.213, Figure 5b).

### Uni- and multivariate analyses for OS

In univariate analysis for OS for all cases, presence of ascites (P = 0.010), GPS 0-1 (P = 0.001), NLR ≥ 3.44 mg/dl (P = 0.012), PMI-high (P = 0.002), and FRPM-high (P = 0.007) were significant factors (Table 4). In multivariate Cox regression analysis, the presence of ascites (P = 0.001), PMI-low (P = 0.021), and FRPM-high (P = 0.028) were independent adverse predictors of OS. HRs and 95% CIs for each parameter are listed in Table 4. In this model, the PMI-high was used as the reference for PMI, and the FRPM-low was used as the reference for FRPM.

### Comparison of baseline data between the FRPM-high and FRPM-low groups

Comparison of baseline data between the FRPM-high and FRPM-low groups showed significant differences in BMI (P = 0.003), serum albumin level (P = 0.011), and GPS score (P = 0.028) (Table 2). Although CRP did not differ significantly between groups, serum albumin was lower and GPS was higher in the FRPM-high group, suggesting a trend toward greater systemic inflammation.

### Comparison of baseline data between PMI-high and PMI-low groups

In the comparison of baseline data between patients with high and low PMI, the distribution of ECOG-PS differed between the two groups (P = 0.006). The proportions of patients with ECOG-PS 0 with high and low PMI were 50.0% (9/18) and 10.5% (2/19), respectively. The proportions of patients with ECOG-PS 1 or 2 in FRPM-high and FRPM-low groups were 50.0% (9/18) and 89.5% (17/19), respectively (Table 3).

## Discussion

We aimed to clarify the prognostic significance of sarcopenia and intramuscular fat infiltration in patients with unresectable PC who received GnP as the first-line chemotherapy. Our findings demonstrated that both lower PMI and higher FRPM were independently associated with shorter OS. Ascites was a significant adverse prognostic factor. Thus, muscle mass and quality, which can be readily evaluated from routine CT scans, are critical determinants of treatment outcomes and survival in patients with advanced PC receiving GnP as the first-line systemic chemotherapy.

Our data align with mounting evidence underscoring the negative prognostic role of sarcopenia in various malignancies. Lisa Lellouche *et al.* reported the association of sarcopenia with poor survival in patients with advanced PC receiving FOLFIRINOX 26. Similarly, Wan *et al.* demonstrated that, in patients with gastrointestinal cancer, decreased skeletal muscle mass predicted worse chemotherapeutic tolerance and overall prognosis 27. Recent meta-analyses restricted to PDAC found that CT-defined sarcopenia is significantly associated with poorer overall survival across resectable and advanced disease settings 28. Our results support these observations by incorporating a measure of muscle quality (i.e., FRPM) into the analysis and confirming its prognostic effect.

Patients with a higher FRPM had a significantly shorter OS, highlighting the emerging role of myosteatosis as a clinically meaningful parameter for cancer prognosis. Interestingly, although the low-FRPM group showed a lower BMI, their overall prognosis was better than that of the high-FRPM group. This finding suggests that low FRPM may represent a relatively preserved muscle phenotype with limited intramuscular fat infiltration, reflecting an earlier or less metabolically impaired stage of cancer progression. In contrast, high FRPM may indicate advanced myosteatosis associated with systemic inflammation and insulin resistance, which are known to worsen survival in cancer patients. Myosteatosis, defined as pathological fat infiltration into the skeletal muscle, is increasingly being recognized as a biomarker of systemic metabolic dysfunction, chronic inflammation, and impaired physical resilience, which are known to contribute to cancer progression and poor treatment outcomes. Tweed *et al.* reported that increased intramuscular fat, measured from CT, is an independent predictor of poor prognosis in patients with stage IV gastric cancer undergoing systemic chemotherapy 29. Although PDAC-specific meta-analyses on myosteatosis are still scarce, our results extend the PDAC sarcopenia literature by incorporating a CT-based marker of muscle quality (FRPM) and suggest that FRPM may be more sensitive than PMI; accordingly, validation in larger, prospective PDAC cohorts is warranted. Consistent with these findings, our study suggests that the FRPM may be a more sensitive marker of cancer-related frailty than PMI alone, reflecting both structural muscle degradation and the metabolic milieu underpinning cancer cachexia and adverse clinical outcomes 30.

Mechanisms linking sarcopenia and myosteatosis to poor cancer outcomes are multifactorial. First, the skeletal muscle is essential for protein storage, energy metabolism, and cytokine regulation, which contribute to host defense and treatment resilience 31. Second, patients with sarcopenia or a higher FRPM are more likely to experience dose-limiting toxicities, early treatment discontinuation, and reduced quality of life 32. Third, both conditions are closely associated with systemic inflammation, as reflected by their correlation with high GPS and low serum albumin 33. These inflammatory and nutritional derangements may fuel a vicious cycle of cachexia, impaired immunity, and tumor progression.

Furthermore, our analysis revealed a significant association between low PMI and poor ECOG-PS scores. This finding supports the notion that imaging-based assessments may provide objective metrics to complement clinician-assigned PS, which can be subjective and less sensitive to subtle declines in functional reserve. Similarly, patients with higher FRPM had lower albumin levels and showed higher systemic inflammation, suggesting that FRPM reflects localized muscle pathology and, broadly, metabolic stress.

The integration of body composition analysis into routine cancer care is becoming increasingly feasible with the advent of automated software tools, such as SYNAPSE VINCENT®. This allows for rapid, reproducible, and standardized measurements of muscle mass and fat infiltration from standard-of-care CT images, thus offering promising avenues for improving prognosis, risk stratification, and personalized treatment planning. Given an aging cancer patient population, sarcopenia before treatment initiation is increasingly emerging as a critical factor in oncology. Older patients are particularly vulnerable to skeletal muscle depletion owing to age-related changes, comorbidities, and decreased physical activity. As such, pretreatment evaluation of sarcopenia using imaging-based metrics, such as PMI and FRPM, can offer valuable insights into a patient's functional reserve and treatment tolerance. Identifying sarcopenia before starting systemic therapy may allow early implementation of supportive interventions, such as nutritional therapy and exercise programs, potentially improving treatment outcomes and preserving quality of life 34. Beyond muscle-related indices, such as PMI and FRPM, systemic inflammatory markers, including GPS and NLR, are significant prognostic indicators in PC. These biomarkers reflect the host's inflammatory and nutritional status and are critically involved in cancer cachexia and mediating treatment responses. GPS, derived from serum CRP and albumin levels, is correlated with survival in patients with advanced PC. Akazawa *et al.* reported the association of elevated GPS with shorter OS in patients undergoing chemotherapy, independent of the tumor burden 33. This suggests that systemic inflammation and malnutrition, captured by the GPS, may be better indicators of host vulnerability than tumor characteristics alone. Because the study by Akazawa *et al.* involved patients with non-small cell lung cancer (NSCLC), the findings may not be directly comparable to pancreatic cancer. Similarly, NLR, calculated as the ratio of neutrophils to lymphocytes in peripheral blood, is a simple yet powerful predictor of poor prognosis. García-Herrera *et al.* demonstrated that a high NLR could independently predict reduced survival in patients with PC, even after adjusting for ECOG-PS and disease stage 35. A high NLR can reflect the imbalance between pro-tumor inflammatory responses and weakened antitumor immunity. Consistent with these findings, our study demonstrated the association of a higher GPS and an elevated NLR with inferior OS, underscoring the clinical relevance of systemic inflammation in PC. Integrating these easily accessible biomarkers with imaging-based assessments, such as PMI and FRPM, may enhance prognostic accuracy and support personalized treatment strategies. Future studies should investigate composite prognostic models that combine these indices in prospective cohorts.

These findings have potential implications for the development of future therapeutic strategies. Patients identified as sarcopenic or with a high FRPM may benefit from early intervention with nutritional supplementation, resistance exercises, and anti-inflammatory therapies. Anamorelin, a ghrelin receptor agonist, holds promise for improving appetite and lean body mass in patients with cancer cachexia 30,36, and its use in conjunction with systemic chemotherapy merits further exploration in patients with PC and sarcopenia. Clinical trials evaluating chemotherapy or novel agents for PC should consider stratifying patients by PMI or FRPM to control for host-related factors that may confound treatment efficacy. Incorporating body composition parameters into the trial design can enhance the interpretability of the results and help identify patient subgroups most likely to benefit from specific interventions.

**Figure 6:**
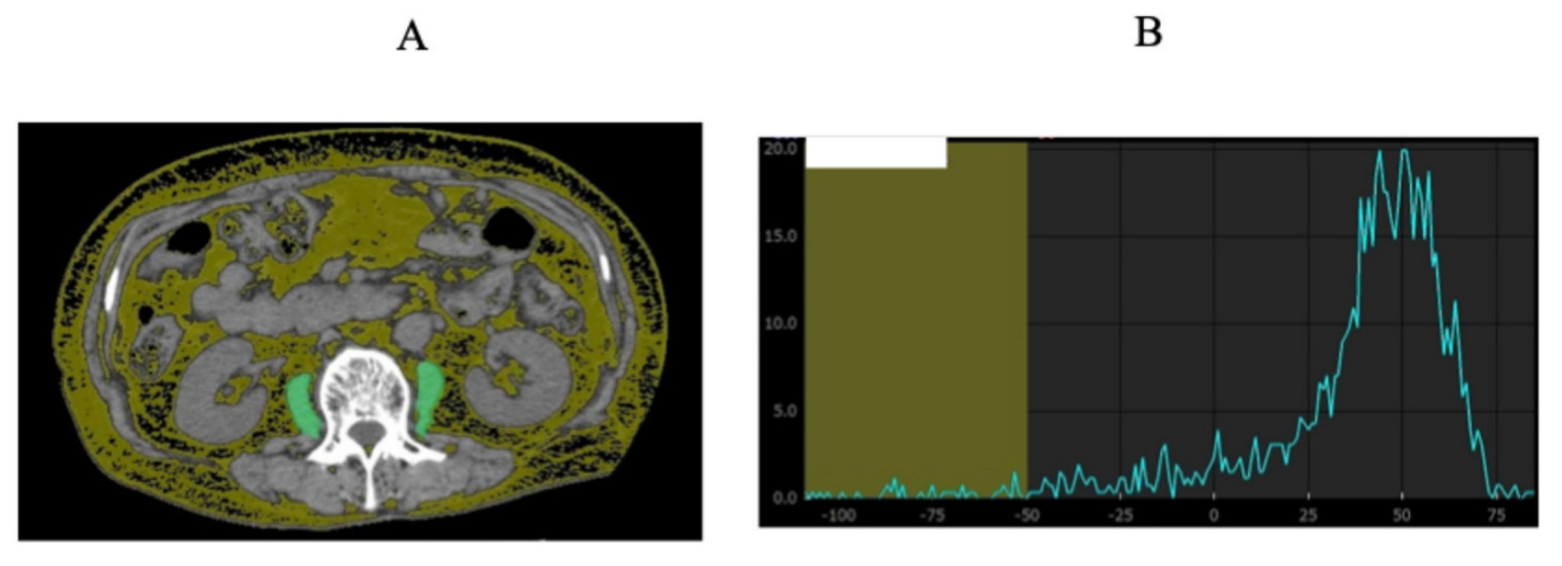
Representative CT images illustrating FRPM measurement and segmentation procedure at the level of the third lumbar vertebra (A). The sum of the areas within the left and right psoas muscles corresponding to fat (CT density: -200 to -50 HU) was quantified using Vincent^®^. The area under the curve corresponding to CT densities below -50 HU and the total area under the curve were calculated, and the fat ratio of the psoas muscle (FRPM) was derived as the proportion of fat area to the total psoas muscle area (B).

This study has some limitations. First, its retrospective design introduces inherent risks of selection bias and limits the ability to establish causality. The decision to initiate GnP therapy and assessment of sarcopenia was not standardized across patients and may have been influenced by clinical judgment. Second, the sample size was small (n = 37), restricting the statistical power to detect subtle associations and limiting the generalizability of the findings. Despite the significance of the findings, they should be interpreted with caution and validated in larger prospective cohorts. Third, PMI and FRPM thresholds were determined based on the median values within the cohort. Although this approach is commonly used in exploratory studies, it may lead to information loss and limit generalizability. Nevertheless, it was selected to enhance clinical interpretability and maintain model stability given the limited sample size. Although ROC curve- or Youden index-derived cutoffs may be informative, such data-driven approaches can be unstable in small heterogeneous cohorts and were therefore not adopted. Consequently, the cutoff values used in this study should be regarded as exploratory, and the lack of external validation or standardized cutoff values may reduce comparability with other studies and limit clinical applicability. The median-based thresholds yielded in our cohort are comparable to those reported in previous Japanese studies supporting internal consistency across domestic cohorts 20. However, international studies have applied different imaging definitions and cutoff strategies for both skeletal muscle quantity and quality, reflecting variability in population characteristics and CT acquisition protocols 29. Therefore, multicenter and international validation using harmonized CT-based methodologies will be essential to establish standardized and broadly applicable reference ranges for these muscle-related parameters. Future studies with larger cohorts should validate our findings using continuous modeling or spline-based analyses. Fourth, although we analyzed imaging-based indices of sarcopenia, direct measures of physical function (e.g., grip strength and gait speed) were not included in our evaluation. The absence of these functional assessments limited our ability to correlate imaging-based indices with objective measures of muscle function. Future prospective studies should incorporate both functional and imaging-based evaluations to provide a more comprehensive assessment of sarcopenia and muscle quality. Fifth, PMI and FRPM were evaluated only at baseline prior to the initiation of chemotherapy, and longitudinal changes were not analyzed because of the retrospective design and limited availability of serial imaging. Future prospective studies with repeated body composition assessments are warranted to elucidate the prognostic impact of temporal changes in muscle mass and quality. Sixth, reproducibility of FRPM measurements was not assessed because all analyses were conducted by a single well-trained researcher. However, the segmentation protocol followed previously validated methods, and representative CT images illustrating the measurement procedure have been added to Figure 6. Seventh, the limited sample size restricted the number of covariates that could be included in the multivariate model. Although key clinical factors such as ECOG performance status, disease stage, relative dose intensity, and second-line therapy are important potential confounders, they were not included because of the small cohort size. Future studies with larger populations are needed to incorporate these variables and validate our findings. Finally, the impact of second-line treatment or best supportive care on survival outcomes is unclear. Variability in subsequent treatments could influence OS and may interact with baseline sarcopenia status.

In conclusion, lower skeletal muscle mass and higher intramuscular fat infiltration were independently associated with poorer OS in patients with unresectable PC treated with GnPs. PMI and FRPM, assessed from routine CT imaging, serve as objective indicators of a patient's nutritional and metabolic conditions. As the cancer patient population continues to age, early identification of sarcopenia and myosteatosis is becoming increasingly important for optimizing treatment strategies. Incorporating body composition analyses into clinical practice may improve risk stratification and guide supportive interventions. Further prospective studies are warranted to validate these findings and assess whether targeting sarcopenia and muscle quality can improve clinical outcomes in this vulnerable population.

## Figures and Tables

**Table 1:** Baseline patient characteristics

	n (%) or Median (range)
Age (years)	70 (49-86)
Sex	
Male	20 (54%)
Female	17 (46%)
ECOG-PS	
0	11 (30%)
1	25 (67%)
2	1 (3%)
Body mass index (kg/m^2^)	20.0 (14.2-32.9)
Ascites	
none	26 (70%)
mild	8 (22%)
moderate	2 (5%)
severe	1 (3%)
Alanine aminotransferase (IU/L)	22 (9-244)
C reactive protein (mg/dL)	0.41 (0.02-17)
eGFR (mL/min/1.73 m^2^)	77 (18-112)
Serum albumin (g/dL)	3.7 (2.3-4.3)
Neutrophil count (/μL)	3887 (1860-21672)
Total lymphocyte count (/μL)	1346 (490-2961)
Glasgow Prognostic Score	
0	8 (22%)
1	17 (46%)
2	12 (32%)
Neutrophil to lymphocyte ratio	3.25 (1.31-21.76)
FRPM (%)	2.55 (0.05-9.01)
PMI (cm^2^/m^2^)	
Male	3.92 (2.11-6.73)
Female	3.57 (2.17-4.49)

ECOG-PS, Eastern Cooperative Oncology Group performance status; eGFR, estimated glomerular filtration rate; PMI, psoas muscle index; FRPM, fat ratio within the psoas muscle.

**Table 2:** Comparison of baseline data between FRPM-high and FRPM-low groups

	n or median		P value
	FRPM-high (n=20)	FRPM-low (n=17)	
Age (years)	66.5	73	0.639
Sex, male/Female	9/11	11/6	0.325
ECOG-PS, 0/1/2	4/15/1	7/10/0	0.226
Body mass index (kg/m^2^)	21.7	18.6	**0.003**
Ascites, none/mild/moderate/severe	14/3/2/1	12/5/0/0	0.206
Alanine aminotransferase (IU/L)	20.5	28	0.939
C reactive protein (mg/dL)	1.07	0.21	0.134
eGFR (mL/min/1.73 m^2^)	70	78	0.126
Serum albumin (g/dL)	3.65	3.9	**0.011**
Glasgow Prognostic Score, 0/1/2	3/7/10	5/10/2	**0.028**
Neutrophil to lymphocyte ratio	7.6	3.19	0.072
PMI-high/low	9/11	9/8	0.630

ECOG-PS, Eastern Cooperative Oncology Group performance status; eGFR, estimated glomerular filtration rate; PMI, psoas muscle index; FRPM, fat ratio within the psoas muscle.

**Table 3:** Comparison of baseline data between PMI-high and PMI-low groups

	n or median		P value
	PMI-high (n=18)	PMI-low (n=19)	
Age (years)	68.5	71	0.679
Sex, Male/Female	10/8	10/9	0.858
ECOG-PS, 0/1/2	9/9/0	2/16/1	**0.006**
Body mass index (kg/m^2^)	20.3	20.0	0.419
Ascites, none/mild/moderate/severe	15/2/1/0	11/6/1/1	0.137
Alanine aminotransferase (IU/L)	21.5	23	0.318
C reactive protein (mg/dL)	0.47	0.41	0.620
eGFR (mL/min/1.73 m^2^)	79.5	68	0.140
Serum albumin (g/dL)	3.7	3.6	0.166
Glasgow Prognostic Score, 0/1/2	2/11/5	6/6/7	0.645
Neutrophil to lymphocyte ratio	3.45	3.24	0.564
FRPM-high/low	9/9	10/9	0.630

ECOG-PS, Eastern Cooperative Oncology Group performance status; eGFR, estimated glomerular filtration rate; PMI, psoas muscle index; FRPM, fat ratio within the psoas muscle.

**Table 4:** Univariable and multivariable analyses for overall survival with Cox proportional hazards models

Covariate	n	Univariable analyses			Multivariable analysis		
		HR	95% CI	P-value	HR	95% CI	P-value
Age ≧ 69 (years), yes/no	20/17	1.048	0.541, 2.030	0.890			
Sex, Male/Female	20/17	1.499	0.764, 2.940	0.239			
ECOG-PS 0, yes/no	11/26	1.714	0.819, 3.587	0.152			
Presence of ascites, yes/no	11/26	0.396	0.189, 0.827	**0.014**	0.243	0.100, 0.587	**0.001**
BMI > 20 kg/m^2^, yes/no	18/19	0.581	0.288, 1.175	0.131			
GPS 0-1, yes/no	25/12	3.284	1.532, 7.039	**0.002**	2.390	0.894, 6.387	0.082
NLR ≧ 3.44, yes/no	17/20	0.400	0.195, 0.818	**0.012**	0.559	0.199, 1.572	0.270
eGFR≧60 ml/min/1.73m2, yes/no	30/7	1.714	0.735, 3.994	0.212			
PMI-high/low	19/18	0.450	0.231-0.902	**0.002**	2.374	1.141, 4.939	**0.021**
FRPM-high/low	20/17	0.365	0.175, 0.760	**0.007**	0.537	0.143, 0.896	**0.028**

ECOG-PS, Eastern Cooperative Oncology Group performance status; BMI, body mass index; GPS, Glasgow prognostic score; NLR, neutrophil-to-lymphocyte ratio; eGFR, estimated glomerular filtration rate; PMI, psoas muscle index; FRPM, fat ratio within the psoas muscle; HR, hazard ratio; CI, confidence interval. Reference categories: PMI-high; FRPM-low. HRs indicate risk category vs. reference category.

## Data Availability

Research data supporting this study are not available in a specific repository. For data access, please contact the corresponding author. The data supporting the findings of this study are not publicly available because they contain information that could compromise the privacy of research participants, but are available from the co-author, T. Y., upon reasonable request.

## Abbreviations

PC: pancreatic cancer
OS: overall survival
GnP: gemcitabine plus nab-paclitaxel
PMI: psoas muscle mass index
FRPM: fat ratio within the psoas muscle
GPS: Glasgow Prognostic Score
NLR: neutrophil-to-lymphocyte ratio
ECOG-PS: Eastern Cooperative Oncology Group performance status
PD: progressive disease
BMI: body mass index
CI: confidence interval
HR: hazard ratio
